# A Putatively Functional Polymorphism in the *HTR2C* Gene is Associated with Depressive Symptoms in White Females Reporting Significant Life Stress

**DOI:** 10.1371/journal.pone.0114451

**Published:** 2014-12-16

**Authors:** Beverly H. Brummett, Michael A. Babyak, Redford B. Williams, Kathleen Mullan Harris, Rong Jiang, William E. Kraus, Abanish Singh, Paul T. Costa, Anastasia Georgiades, Ilene C. Siegler

**Affiliations:** 1 Department of Psychiatry and Behavioral Sciences, Duke University School of Medicine, Durham, North Carolina, United States of America; 2 Department of Sociology, The University of North Carolina at Chapel Hill, Chapel Hill, North Carolina, United States of America; 3 Carolina Population Center, The University of North Carolina at Chapel Hill, Chapel Hill, North Carolina, United States of America; 4 Duke Molecular Physiology Institute and Division of Cardiology, Duke University School of Medicine, Durham, North Carolina, United States of America; UTHSCSH, United States of America

## Abstract

Psychosocial stress is well known to be positively associated with subsequent depressive symptoms. Cortisol response to stress may be one of a number of biological mechanisms that links psychological stress to depressive symptoms, although the precise causal pathway remains unclear. Activity of the x-linked serotonin 5-HTR2C receptor has also been shown to be associated with depression and with clinical response to antidepressant medications. We recently demonstrated that variation in a single nucleotide polymorphism on the *HTR2C* gene, rs6318 (Ser23Cys), is associated with different cortisol release and short-term changes in affect in response to a series of stress tasks in the laboratory. Based on this observation, we decided to examine whether rs6318 might moderate the association between psychosocial stress and subsequent depressive symptoms. In the present study we use cross-sectional data from a large population-based sample of young adult White men (N = 2,366) and White women (N = 2,712) in the United States to test this moderation hypothesis. Specifically, we hypothesized that the association between self-reported stressful life events and depressive symptoms would be stronger among homozygous Ser23 C females and hemizygous Ser23 C males than among Cys23 G carriers. In separate within-sex analyses a genotype-by-life stress interaction was observed for women (p = .022) but not for men (p = .471). Homozygous Ser23 C women who reported high levels of life stress had depressive symptom scores that were about 0.3 standard deviations higher than female Cys23 G carriers with similarly high stress levels. In contrast, no appreciable difference in depressive symptoms was observed between genotypes at lower levels of stress. Our findings support prior work that suggests a functional SNP on the *HTR2C* gene may confer an increased risk for depressive symptoms in White women with a history of significant life stress.

## Introduction

The 5-HTR2C serotonin receptor has been extensively investigated. The role it plays with respect to numerous critical neural circuits within the brain makes it a promising candidate for genetic association studies of behavior and psychological disorders. The *HTR2C* gene, which is x-linked, has been associated with psychological disorders, feeding behavior, antipsychotic-induced side effects [1], and greater stress-induced mesoaccumbal dopamine release sensitivity [2]. An *HTR2C* variant that has received considerable attention is rs6318, a nonsynonymous coding single nucleotide polymorphism (SNP). This SNP variant (G to C) leads to a substitution of serine for cysteine at codon 23 (Cys23Ser). There is evidence that the Ser23 C allele is constitutively more active than the Cys23 G allele, with allele-specific analysis of the receptor showing functional differences in the activity of the Ser23 C minor allele relative to the Cys23 G allele [3]. Some studies, however, have failed to find in vitro differences between Ser23 and Cys23 variants [4]; thus it is possible that the N-terminal region of the receptor containing this residue is not part of the mature protein in vivo [5]. The frequency of the Ser23 C allele is approximately 17% in unrelated Whites (http://www.1000genomes.org/).

The 5-HTR2C receptor plays a key role in the mediation of stress-induced hypothalamic-pituitary-adrenal (HPA) axis activation by the release of serotonin in the central nervous system (CNS) [6]–[8]. We have recently shown in two independent samples [9], [10] that individual variation in rs6318 in adult humans is associated with cortisol response to a laboratory stress recall task. Specifically, in a sample of adult males, Ser23 C allele carriers exhibited two-fold larger increases in plasma cortisol during recall of situations in which they experienced anger or sadness compared to those carrying the Cys23 G allele [9]. More recently, we validated and extended this finding in a sample that also included women [10]. Studying the ecological validity of laboratory cortisol responses to stress, Kidd et al. [11] has demonstrated that cortisol responses assessed during laboratory stress are positively associated with cortisol area under the curve (AUC) levels during the day, supporting the generalizability of laboratory measure to those made in the field.

Cortisol has been proposed as an important causal component in the development of depressive symptoms. In a recent review, Herbert [12] concluded that the role of cortisol in the genesis of depressive symptoms is multifaceted, and that genetic variation and environmental factors magnify this risk. Herbert also notes that this role starts prenatally, continues throughout the lifespan, and is influenced by the timing of environmental factors. Herbert [12] also provides evidence suggesting that there may be important gender differences with respect to the relation between cortisol and depression, perhaps due in part to the influence of estrogens. In related work, Cooke and Weathington [13] hypothesize that females have more reactive stress response systems, which may dispose them to develop depression at higher rates than males. Finally, in a study of 6,126 White men and women referred for diagnostic cardiac catheterization, we reported that over a 7-year follow-up period males hemizygous for Ser23 C and females homozygous for Ser23 C allele had an increase in cardiovascular disease event (death and/or MI) risk [hazard ratio (HR) = 1.38] compared to those carrying the Cys23 G allele [14]. One possible explanation for this finding is that variation in rs6318 might be ultimately related to cardiovascular events by way of HPA-axis hyperactivity. In the same study, we observed that the association was somewhat stronger in females (HR = 1.62) compared to males (HR = 1.23).

Given the evidence that rs6318 might moderate HPA-axis response to acute stress, and the putative role of HPA-axis response in the development of depressive symptoms, we hypothesized that variation in rs6318 would also potentiate the association between life stressors and depressive symptoms. Specifically, we expected that there would be little difference in depressive symptoms between genotype groups at low levels of life stress. However, at higher levels of life stress, we predicted that homozygous Ser23 C women and hemizygous Ser23 C allele men would have higher ratings of depressive symptoms compared to Cys23 G carriers at similar levels of stress. To date, no human studies appear to have examined this possibility with respect to rs6318. Moreover, the few studies that have examined rs6318 have in general relied on clinical or convenience samples. In contrast, the present study uses data from a study with a rigorously planned and executed nationally representative sample of the population of the United States. Sex by genotype cell frequencies among Hispanic, Asian, and Native American were insufficient for reliable analysis, and the Black sample did not meet the Hardy-Weinberg assumption. We therefore limited the present analysis to the subsample of White study participants. We also examined the potential role of gender with respect to this genotype by life stress interaction.

## Methods

### Participants

The Add Health study uses a nationally representative sample of approximately 15,000 young adults to investigate health and health-related behaviors during adolescence and into young adulthood. The study was reviewed and approved by the IRB at The University of North Carolina at Chapel Hill. Written consent was obtained for all data collection. The participants have been followed from grades 7 through 12 in 1995 through early adulthood in 2008–09 in 4 waves of data collection [15]. As noted above, due to limited cell frequencies among race and gender groups, as well as lack of Hardy-Weinberg equilibrium for Blacks, we also limited the sample to White men and women. Cases that did not meet genotyping quality control standard also were eliminated from the analysis. In addition, the full Add Health dataset includes some clusters of genetically-related individuals. In these cases, we excluded all but the case with the lowest study identification number in a given family cluster. These exclusions yielded a final, sample of 5,623 White individuals.

### Measures

#### Genotyping

Saliva was collected for genotyping using the Oragene collection method (Oragene, DNAgenotek, Ottawa, Ontario, Canada). Genomic DNA was isolated from the Oragene^TM^ solutions using ZymoResearch (Irvine, CA). Silicon-A plates and the DNA was quantified using Picogreen® (Invitrogen, Carlesbad, CA) fluorescence. The mean (standard deviation) and range of the isolated DNA yield was 33 (25) and 0 to 400 µg. SNP assays were done on either an Applied Biosystems TaqManOpenArray or Illumina BeadXpress GoldenGate platform. SNP genotyping was performed according to company-supplied protocols (Applied Biosystems, Inc.; Illumina, Inc.). The rs6318 SNP was genotyped simultaneously along with other SNPs. Participants with a missing SNP genotype rate >10% were excluded according to laboratory quality control standards. The genotyping rate for rs6318 was greater than 95%. The kappa coefficient comparing split sample assays on 48 randomly selected participants was 1.0. Because rs6318 is located on the X chromosome, the Hardy-Weinberg equilibrium (HWE) test was conducted only in females and the genotype distribution was consistent with HWE (P = 0.192) among Whites.

#### Demographic Measures

Age was assessed at Wave IV. Race was constructed from a series of self-report interview queries at Wave I.

#### Stressful life events index

Stressful life events were quantified using an additive index of stressful life events (SLE) developed for the Add Health study, which was assessed at the Wave IV in-person interview. Briefly, the items (see S1 Appendix) were adapted from a measure developed by Ge et al. [16] using a priori criteria [17]. Only events of sudden onset within the 12 months preceding the interview were included [17]. The index includes 35 items from a wide range of areas (e.g., family, romantic and peer conflicts, academic problems, involvement or exposure to violence, death of family or friends). The original stress index included suicidal gesture as a life stressor. This item was removed for the present study in order to avoid confounding the stress score with the depressive symptom response variable. The final score is derived by a simple unit-weighted sum of stressful events, with a possible range of 0–35.

#### Depressive symptoms (brief CES-D)

Depressive symptoms were assessed at the Wave Iv interview using a nine-item scale culled from the conventional 20-item Center for Epidemiologic Studies Depression Scale (CES-D) [18]. The original 20-item CES-D is composed of questions on a variety of symptoms of depression, which cluster into four factors: Somatic-Retarded Activity, Depressed Affect, Positive Affect and Interpersonal Relations [18], [19], and has well-documented psychometric properties in both Whites and Blacks [20]. The initial measurement waves in Add Health used a 19-item version of the CES-D, but the scale was reduced to 9 items in later waves in order to reduce participant burden (see S1 Appendix). Data from the earlier waves shows that the 9-item scale used in the present study correlated highly with the 19-item scale (*r* = .91 and .92 at Waves I and II, respectively). Individual items are coded on a four-point scale to indicate the frequency of symptoms occurring during the past week, ranging from never or rarely (0) to most or all of the time (3). The interview questions were framed to assess symptoms in the past 7 days. The reliability of the nine-item scale at Wave IV was adequate, with a standardized alpha of .83 for Whites.

#### Antidepressant medication

Antidepressant medication status was assessed at the Wave IV in-home interview, and includes any recognized class of antidepressant medication, including serotonin selective reuptake inhibitors (SSRIs), tricyclic antidepressants (TCAs), norepinephrine selective reuptake inhibitors (NSRIs), and other miscellaneous antidepressants.

### Statistical Analyses

Our primary analyses were conducted using Proc Mixed in SAS 9.3 (SAS Institute, Cary, NC). We conducted separate analyses for men and women, predicting CES-D score with age, antidepressant use (no/yes), life stress index, rs6318 genotype as a 3-level factor, and a life stress index by rs6318 product interaction term. For mixed model analyses, the CES-D score was transformed using the square root in order to better meet model residual assumptions. All models included the sampling cluster term (PSUSCID) as a random effect, and post-stratification weights (GSWGT4_2). As a follow-up to significant interactions, we conducted “slice” tests available in Proc Mixed, which compared the genotype groups at selected low and high values of the stress index score. We also explored the possibility of nonlinearity between the stress index and depressive symptoms using restricted cubic splines in the rms package in R 3.03 (http://cran.us.r-project.org/). For the latter analysis, the stress index was modeled as a 3-knot restricted cubic spline within a linear model, with the model including post-stratification weights and Huber-White standard errors to account for sample clustering. In order to more formally assess the comparison in associations between men and women, we also estimated a mixed model with men and women combined in a single sample predicting CES-D score by gender, antidepressant use, rs6318 genotype, two-way interaction terms of gender by rs6318, stress by gender, and stress by rs6318, and a three-way interaction term, gender by stress by rs6318. As rs6318 is x-linked, interactions involving the genotype and sex can be somewhat obscure in interpretation. We therefore removed heterozygous women from the sample for this latter analysis of the three-way interaction in order to facilitate interpretability.

We conducted an *a priori* power analysis using the Quanto software (http://biostats.usc.edu/Quanto.html) to determine the effect sizes that would be detectable given the known available sample sizes of women and men. Among women, we estimated that with a sample size of 2,712, assuming a genetic main effect of R^2^ = 0.001, a stress main effect of R^2^ = 0.01 and alpha of 0.05, we would have 80% power to detect an interaction with an effect size of R^2^ = 0.003. Under the same assumptions, the male sample size of 2,366 also would yield 80% power to detect an effect size of R^2^ = 0.003.

## Results

Sample characteristics for women and men are displayed in Tables 1 and 2, respectively. The observed range of stress index scores was 0 to 14 (possible range, 0–35), with 91% of the sample reporting 4 or fewer stressful life events. The most frequently reported stressor for both men and women was skipping medical care due to financial reasons (see S1 Appendix). Being unable to pay bills was the second most frequent stressor for women and third most frequent for men, while being the victim of physical assault was the second most frequent for men and third most for women. We observed no differences in background characteristics across genotype in either sex other than a difference on the stress index in women, where G carriers had higher median stress index scores than C homozygotes. Further examination of this difference revealed that the maximum stress index value among C homozygous women was 6, while the range among G carriers extended to 14. We explore this difference further in sensitivity analyses described below. About 12% of women and 6% of men reported taking some form of antidepressant medication. Among the women using antidepressant medication, 69% were using selective serotonin reuptake inhibitors (SSRIs), 15% were taking serotonin–norepinephrine reuptake inhibitors (SNRIs), 3% tricyclic antidepressants (TCAs), 1% tetracyclic antidepressants, and 12% miscellaneous antidepressant medication. For men, 62% reported taking an SSRI, 13% SNRIs, 4% TCAs, 2% tetracyclic antidepressants, and 18% miscellaneous types.

**Table 1:** Descriptive Statistics by rs6318: Women.

	N	C/CN = 86	G/CN = 738	G/GN = 1888	P-value for genotype comparison
Age, Years	2712	27.6 **28.8** 30.5	27.3 **28.9** 30.3	27.4 **28.7** 30.1	0.708
Stress	2712	0 **1** 2	0 **2** 3	0 **2** 3	0.001
CES-D	2712	2.25 **5.00** 7.00	2.00 **4.50** 8.00	2.00 **4.00** 8.00	0.899
Antidepressant	2712	14% (12)	11% (78)	12% (218)	0.577
Married	2711	69% (59)	60% (440)	64% (1201)	0.082
Annual Income	2572				0.821
<35 K		27% (22)	31% (215)	32% (564)	
35–62.5 K		43% (36)	38% (267)	37% (668)	
>62.5 K		30% (25)	31% (219)	31% (556)	
Education	2712				0.763
<HS		5% (4)	5% (40)	6% (114)	
HS		17% (15)	13% (95)	13% (250)	
Some College		41% (35)	46% (338)	43% (818)	
College		26% (22)	20% (151)	22% (424)	
Postgrad		12% (10)	15% (114)	15% (282)	
Alcohol Use	2705				0.330
None		27% (23)	24% (177)	24% (443)	
Light		66% (57)	67% (495)	69% (1296)	
Moderate		2% (2)	3% (22)	3% (62)	
Heavy		1% (1)	3% (20)	1% (22)	
Very Heavy		3% (3)	3% (24)	3% (58)	
Regular Exercise	2710	87% (75)	85% (625)	85% (1604)	0.823
Smoking	2702	28% (24)	26% (195)	27% (500)	0.940

*a* **b** *c* represent the lower quartile *a*, the median *b*, and the upper quartile *c* for continuous variables.

*N* is the number of non–missing values.

Numbers after percents are frequencies.

Kruskal-Wallis test for continuous variable; Pearson chi-square test used for frequencies.

**Table 2:** Descriptive Statistics by rs6318: Men.

	N	C/-N = 360	G/-N = 2006	P-value for genotype comparison
Age, Years	2366	27.9 **29.0** 30.3	27.6 **29.1** 30.4	0.708
Stress	2366	1 **2** 3	1 **2** 3	0.820
9-item CES-D	2366	2 **4** 6	2 **4** 7	0.290
Antidepressant Use	2366	5% (19)	6% (112)	0.815
Married	2364	49% (176)	52% (1041)	0.285
Annual Income	2255			0.680
<35 K		26% (90)	29% (551)	
35–62.5 K		40% (135)	38% (730)	
>62.5 K		34% (115)	33% (634)	
Education	2366			0.265
<HS		8% (30)	9% (173)	
HS		23% (84)	18% (371)	
Some College		43% (155)	45% (910)	
College		18% (65)	19% (372)	
Postgrad		7% (26)	9% (180)	
Alcohol Use	2352			0.472
None		22% (79)	19% (378)	
Light		58% (209)	63% (1256)	
Moderate		4% (14)	4% (76)	
Heavy		4% (13)	3% (69)	
Very Heavy		12% (45)	11% (213)	
Regular Exercise	2365	90% (323)	88% (1757)	0.201
Smoking	2351	31% (109)	30% (607)	0.973

*a* **b** *c* represent the lower quartile *a*, the median ***b***, and the upper quartile *c* for continuous variables.

*N* is the number of non–missing values.

Numbers after percents are frequencies.

Kruskal-Wallis test for continuous variable; Pearson chi-square test used for frequencies.

**Figure 1:**
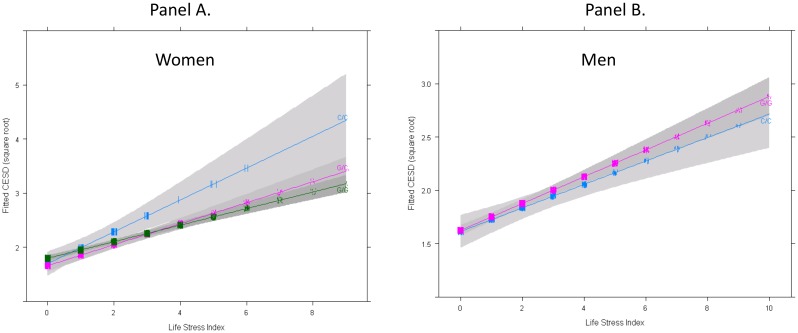
Interaction between the stress index and rs6318 genotypes predicting depressive symptom scores among White women (Panel A) and White men (Panel B). Estimates are adjusted for antidepressant use and age. Shaded gray areas represent 95% confidence bands generated using the Huber-White estimator. Tick marks represent case density. The sample was comprised of 86 C/C, 738 G/C, and 1,888 G/G women, and 360 C/- and 2,006 G/- men. The interaction term was statistically significant for women (p = .022), but not for men (p = .471).

In the separate within-sex mixed models, we observed a life stress by genotype interaction predicting CES-D among females (p = .022) but little evidence of this interaction in males (p = .471). As expected, antidepressant use was strongly associated with CES-D scores for women (b = 0.47, p<.001) and men (b = 0.56, p<.001). The gender differences in the two-way interaction were supported by a sex by stress by genotype interaction in a model that combined the male and female data (p = .025). The spline analysis suggested insufficient evidence for a nonlinear association between the stress index and depressive symptoms (p = .250). Thus, we maintained the linear specification of this association in the final model. Fig. 1 displays the form of the stress by genotype interaction for women and men. At low stress, the genotype groups differed very little on CES-D score. At the higher stress level, however, women homozygous for the C allele had higher CES-D scores than either group with the G allele. Testing simple effects using model-predicted values, when stress index scores were 1, the predicted CES-D score for C/Cs was only 0.15 points higher than G/Cs (p = .154) and .03 points higher than G/Gs (p = .773). When stress index scores were 3, however, the predicted CES-D scores for C/Cs were .34 points higher than G/Cs (p = .030) and .30 points higher than G/Gs (p = .048). The standard deviation of the square root transformed CES-D score was .97. Thus, the standardized differences between C/Cs and the other genotypes when the stress score was 3 are roughly 0.3.

As noted earlier, we observed that the median life stress score was lower among C/C women (median SLE = 1) compared to G/Cs and G/Gs (median = 2) and also that the range of life stress scores among G carriers extended beyond the range for C/Cs. No C/C women scored above 6 while 49 G/G and G/C women had stress scores ranging from 7 to 14. To evaluate the possible role of these higher stress G/G women in the regression results, we re-estimated the primary model using only participants with life stress scores 6 or lower. Truncating the sample in this manner resulted in identical median stress scores of 1 in C/C and G/G women. In within-sex analyses, the stress by genotype interaction predicting CES-D among women remained intact (p = .011). By way of descriptive comparison, the raw median CES-D score for the 43 highly stressed (stress indexes >6) G/G women was 3.2 (interquartile range = 1.4), a level similar to C/C women (median = 3.0, interquartile range = 1.1) with moderate stress indexes (between 4 and 6). In other words, C/C women with moderately high stress scores had depressive symptom levels comparable to G/G reporting very high levels of life stress.

Two anonymous reviewers also suggested adjustment in the statistical model for additional variables, including Body Mass Index (BMI), marital status, and corticosteroid medication use. Adding these variables to our primary within sex models did not materially alter the results. For women, adjusting for these variables actually reduced the standard error of the stress by SNP interaction test, resulting in a smaller p-value compared to the primary model (p = .012). The stress by SNP interaction for men remained non-significant (p = .450). Reviewers also requested univariate associations among the CESD, stress index, and antidepressant medication; and output from Mixed Models. We present these correlations in S2 Appendix, and results of Mixed Models in S3 Appendix.

## Discussion

Our hypothesis was supported, but only for the female sample, such that the association between stress and depressive symptom scores depended on the rs6318 genotype. Specifically, females homozygous for the Ser23 C allele who reported higher levels of life stress had depressive symptom scores that were about 0.3 standard deviations higher than female Cys23 G carriers with similar high stress levels. In contrast, depression levels were essentially equivalent across genotype among women reporting lower levels of life stress. Our prior work with rs6318 [10], [14], [21] to date suggests that women who carry the Ser23 C allele are significantly more likely than G allele carriers to experience an increased cortisol response and negative affect to an acute stressor, and perhaps increased risk of cardiovascular disease-related events. Specifically, we have shown in two independent samples that the cortisol response to a stress task was two-fold larger among those with the Ser23 C allele compared to those carrying the Cys23 G allele–providing evidence for the proposition that the 5-HT2C receptor plays a key role in the activation of the HPA axis by acute stress. Interestingly, in the present analyses homozygous Ser23C women also reported fewer stressful life events than Cys23G carriers. There is no readily available explanation for this difference, but we might speculate that Ser23C homozygous women might be prone to avoiding situations that have the potential to elicit stress [22]. Most importantly, our sensitivity analyses suggested that this difference did not explain the interaction between stressful life events and rs6318. Indeed, even when the influence of higher stress levels among Cys23G carriers was removed, predicted depressive symptoms scores remained highest among Ser23 CC women.

Demonstrating a stress by genotype interaction for females but not for males is consistent with at least some, but not all of our prior work [14], and is also supported by additional biological evidence. For example, both animal and human models show that females exhibit higher levels of glucocorticoids (cortisol or corticosterone) as compared to males [23], [24]. Post-pubertal females show an excess of major depressive disorder which could be, in part, a result of increased cortisol levels not observed in men [12]. Sex hormones are implicated in developmental effects during fetal life, as well as in activating effects of the HPA-axis later on, and such a mechanism may contribute to the higher prevalence of mood disorders in women as compared to men [13]. In a recent extensive review of the sex specific effects of child abuse, Cooke and Weathington [13] posit that sex differences in how the social world is perceived may be responsible for the increased risk of depression in women. These sex differences, with regard to the increased perception of threat and empathetic emotional responding among females, may be the result of the effects of sex steroid hormones during perinatal and pubertal development and may not manifest until adulthood [13]. Another possible reason that the interaction was observed only in women is that men have only one copy of the Ser23 C allele, compared to two copies among the women who exhibited the highest depressive symptom scores at higher levels of stress. In addition to possible biological mechanisms, learned gender roles in Western industrialized societies also may play a part in the differences we observed between men and women. For example, some [25] suggest that men may be more likely to cope with stress by actively engaging in distracting activities, while women may tend to take a less active strategy and engage in ruminative thinking, the latter posing a risk for developing depressive symptoms.

The association between rs6318 and cortisol response may provide insight into the observed association of the Ser23 C variant with depression [1], [26]. It has also been proposed that early adversity may induce chronically high cortisol levels, in turn increasing the risk for major depressive disorder later in life [27]. Individuals who are sexually or physically abused in childhood experience distinctly enhanced activation of the HPA axis as adults [12]. Furthermore, even among individuals who are not currently depressed, enhanced adrenocorticotropic hormone (ACTH) responses are observed when exposed to a standardized psychosocial stressor, while those who are currently depressed show the largest increases in ACTH and cortisol secretion [28]. Such findings suggest that the HPA-axis hyperactivity associated with depression might be the result of prolonged neurobiological abnormalities that predispose individuals to this disorder. The failure to account for the physiological challenge of life stress may therefore help explain some of the inconsistencies with regard to the presence of HPA axis hyperactivity in depression [29]. The present findings suggest the Ser23 C allele may modify this increased vulnerability with regard to heightened stress and cortisol levels in response to psychosocial stress, leading to depression in adulthood among White women.

Human diseases and psychological disorders generally result from a complex interaction between genetic profiles and environmental exposures throughout the lifespan. Genetic differences also appear to influence individual differences in the physiological response to exposure to commensurate environmental stimuli. Differences in response may, in the long run, induce pathophysiological changes that lead to disease states, such as affective disorders. The present findings point to the potential advantage of considering effects of environmental stress and gender with regard to associations among genes and behavioral traits. For example, a prior finding in the Add Health study [30] reported that allelic variation in 5-HTTLPR interacted with perceived stress to predict depressive symptoms among females, but not males. Specifically, women with the short/short variant who also reported high ratings of stress were more likely to report depressive symptoms compared to women with the long allele. Similarly, a 5-HTTLPR by stress group by gender interaction was a significant predictor of depressive symptoms in two independent samples [31]. For females, the short allele (lower expression) was associated with increased ratings of depressive symptoms in those with higher stress, as compared to those with the long (lower expression) allele; whereas, in males, the long allele combined with a stressor, was associated with higher depression scores as compared to those in the non-stressor group and those with the short allele. In our own work on stress and the serotonergic system, we reported that a genotype (5-HTTLPR) by gender interaction also predicted negative affect ratings during intravenous infusion of L–tryptophan. In that study, females with a low-expression genotype and males with a high expression genotype showed the greatest increases in negative affect during the tryptophan infusion [32]. Thus, the evidence mounts that gene by stress by gender interactions should always be examined when studying the effects of a particular genotype with regard to psychological disorders, and perhaps other diseases as well.

An important limitation of the current study is that we were unable to examine the effects of rs6318 on depressive symptoms in racial groups other than Whites due to sparse cell counts or the failure to meet the HWE assumption. Thus, we cannot know whether the present findings would generalize to groups other than self-identified Whites. However, the reliability of the findings for Whites is bolstered by the carefully constructed sampling design intended to still capture representative data from that ethnic group. The present sample considers only a relatively narrow slice of the lifespan, young adulthood, and does not speak to the nature of these associations during other periods of the lifespan. The use of a limited interview measure of depression rather than the “gold standard” structured clinical interview also may be seen by some as a limitation. Such compromises, of course, are often necessary to carrying out a study as large and wide-ranging as Add Health. As noted earlier, however, the depression measure displayed adequate internal consistency, and also appears to have face valid content for the affective component of depressive symptoms. Moreover, the depression measure behaved in ways that one would expect, with antidepressant and life stress use strongly associated with symptoms. The Add Health Study also contains measures of suicide and self-reported diagnosis of depression. We elected to not use these measures due to concern regarding reliability, but also because they were assessed in a manner that would have made it possible for the stressful event to occur after the window for the depression diagnosis or suicidal gesture or ideation. Despite the limited number of items, the CES-D was framed to assess depressive symptoms during week preceding the interview, thus making it very unlikely that a stressor occurred after the report of depressive symptoms. Although stressful life events were recalled retrospectively, the inventory assessed only objective events (e.g. divorce, job loss) that were of sudden onset within the past 12 months, and only whether the event occurred or not, rather than the level of perceived stress the event incurred. Recall bias due to affect is therefore likely minimal in comparison to studies that have individuals recall many types of events across the lifespan or that attempt to evaluate the severity of the stressful event. In addition, ancestry markers are not yet available for the Add Health study and we were therefore unable to evaluate the extent to which confounding due to stratification may have influenced the present results. Finally, although we are confident in the robustness of our present results in this particular sample, we emphasize the need for replication of the present finding in new samples, preferably over a range of population characteristics and assessment methods.

The above limitations notwithstanding, it is generally accepted that cortisol plays a multifaceted role in the onset and development of depression, a role that likely varies by gender, and also is perhaps influenced by genetic predisposition [12]. Given our previous findings that the Ser23 C variant influences the cortisol stress response in two independent samples [9], [10] and clinical course in CHD patients [14] the current findings suggest that this SNP interacts with stress in women resulting in carriers exhibiting increased symptoms of depression. A next step in studying rs6318 might include the use of further *in vitro* or *in vivo* tests using pharmacological probes to further evaluate the genetic contribution of the cortisol response. For example, 5-HTR2C sensitivity might be manipulated with an agonist or antagonist, and the differential physiological or psychological response to stress recorded as a function of genotype and the presence or absence of the probe. In the longer term, understanding how rs6318 influences cortisol and mood responses to acute and chronic stress could ultimately guide intervention against both exaggerated cortisol reactivity and depressive symptomology. Further work also might advance the understanding of why both depressive symptoms and cortisol dysregulation have been linked with an increased risk of coronary artery disease. Interventions that reduce cortisol response to acute stress and depressive symptoms in women exposed to chronic stress could be evaluated as a means of reducing risk of developing CHD and/or improving prognosis once disease is present. Finally, at a more general level, when a phenotype is known to be multi-determined and complex as in the case of depressive symptoms, the study of a single genetic variant provides only one very small piece of a complex puzzle. Ultimately, a finding such as the present one must be embedded in a more complete description of the underlying causal system, including not only many genes, but also the greater psychosocial and developmental context. A critical challenge in adequately describing such a system will necessitate very large amounts of data with reliable measures. At present, our group has begun to undertake an investigation of the fuller architecture that might surround the isolated hints that single variant studies have produced.

## Supporting Information

S1 Appendix **Items from Stress Index and modified Center for Epidemiological Studies Depression Scale.** (CES-D).
(DOCX)

S2 Appendix **Spearman correlations among study variables.**
(DOCX)

S3 Appendix **Mixed model results for women and men.**
(DOCX)
