# Supplementary material for: A Putatively Functional Polymorphism in the HTR2C Gene is Associated with Depressive Symptoms in White Females Reporting Significant Life Stress
Source: PLoS One. 2014 Dec 16;9(12):e114451. doi: 10.1371/journal.pone.0114451 (PMC4267787; doi:10.1371/journal.pone.0114451)
Supplement: S1 Appendix — Items from Stress Index and modified Center for Epidemiological Studies Depression Scale. (CES-D). (DOCX) [file pone.0114451.s001.docx]

## Appendix S1

Women

|  | Stressor | Count | % of group |
| --- | --- | --- | --- |
| 1. | Skipped Medical Care For Financial Reasons | 678 | 25.0 |
| 2. | Could Not Pay Bills | 670 | 24.7 |
| 3. | Was the Victim of Physical Assault | 496 | 18.3 |
| 4. | Witnessed Violence | 399 | 14.7 |
| 5. | Was Diagnosed with a Serious Disease | 358 | 13.2 |
| 6. | Contracted a Sexually Transmitted Disease | 346 | 12.8 |
| 7. | Had a Serious Injury | 329 | 12.1 |
| 8. | Was in a Motor Vehicle Accident | 257 | 9.5 |
| 9. | Was Physically Abused in a Romantic Relationship | 252 | 9.3 |
| 10. | Had a Romantic Relationship that Ended | 243 | 9.0 |
| 11. | Had a Friend/Relative Committed Suicide | 208 | 7.7 |
| 12. | Lost a Sibling | 207 | 7.3 |
| 13. | Became Disabled | 129 | 4.8 |
| 14. | Lost Utility Service for Financial Reasons | 111 | 4.1 |
| 15. | Was Arrested | 72 | 2.7 |
| 16. | Was Sexually Abused in a Romantic Relationship | 71 | 2.6 |
| 17. | Lost A Friend/Relative to Suicide | 66 | 2.3 |
| 18. | Was in a Physical Fight | 61 | 2.3 |
| 19 | Lost a Job | 50 | 1.8 |
| 20. | Lost a Parent | 46 | 1.7 |
| 21. | Was Put in Jail | 43 | 1.6 |
| 22. | Had an Still Birth or Miscarriage | 33 | 1.2 |
| 23. | Had a Criminal Conviction as an Adult | 18 | 0.7 |
| 24 | Had an Unwanted Pregnancy | 16 | 0.6 |
| 25. | Was Deployed in a Combat Zone (military) | 12 | 0.4 |
| 26. | Witnessed Death in War (military) | 8 | 0.3 |
| 27. | Was Raped | 7 | 0.3 |
| 28. | Injured in Combat (military) | 4 | 0.2 |
| 29. | Had an Abortion (partner) | 4 | 0.2 |
| 30. | Was Evicted | 3 | 1.1 |
| 31. | Was in Full Time Active Duty Service (military) | 2 | 0.1 |
| 32. | Lost a Child | 2 | 0.1 |
| 33. | Engaged Enemy in a Fire Fight (military) | 1 | 0.0 |
| 34. | Gave Up a Baby for Adoption | 0 | 0.0 |
| 35. | Kill Someone in Combat (military) | 0 | 0.0 |

Men

|  | Stressor | Count | % of group |
| --- | --- | --- | --- |
| 1. | Skipped Medical Care For Financial Reasons | 618 | 26.1 |
| 2. | Was the Victim of Physical Assault | 528 | 22.3 |
| 3. | Could Not Pay Bills | 506 | 21.4 |
| 4. | Had a Serious Injury | 441 | 18.6 |
| 5. | Was Physically Abused in a Romantic Relationship | 337 | 14.2 |
| 6. | Witnessed Violence | 306 | 12.9 |
| 7. | Had a Romantic Relationship that Ended | 251 | 10.6 |
| 8. | Was Diagnosed with a Serious Disease | 246 | 11.2 |
| 9. | Was in a Motor Vehicle Accident | 235 | 9.9 |
| 10. | Was in a Physical Fight | 215 | 9.1 |
| 11. | Had a Friend/Relative Committed Suicide | 149 | 6.3 |
| 12. | Was Arrested | 144 | 6.1 |
| 13. | Lost a Sibling | 142 | 6.0 |
| 14. | Was Deployed in a Combat Zone (military) | 126 | 5.3 |
| 15. | Contracted a Sexually Transmitted Disease | 111 | 4.7 |
| 16. | Lost Utility Service for Financial Reasons | 103 | 4.4 |
| 17. | Was Put in Jail | 96 | 4.1 |
| 18. | Witnessed Death in War (military) | 95 | 4.0 |
| 19 | Had a Criminal Conviction as an Adult | 75 | 3.2 |
| 20. | Engaged Enemy in a Fire Fight (military) | 73 | 3.1 |
| 21. | Was Sexually Abused in a Romantic Relationship | 67 | 2.8 |
| 22. | Kill Someone in Combat (military) | 62 | 2.6 |
| 23. | Lost A Friend/Relative to Suicide | 53 | 2.4 |
| 24 | Lost a Job | 53 | 2.2 |
| 25. | Became Disabled | 41 | 1.7 |
| 26. | Lost a Parent | 38 | 1.6 |
| 27. | Injured in Combat (military) | 23 | 1.9 |
| 28. | Was Evicted | 21 | 0.9 |
| 29. | Had an Unwanted Pregnancy | 21 | 0.9 |
| 30. | Had an Still Birth or Miscarriage | 17 | 0.7 |
| 31. | Was in Full Time Active Duty Service (military) | 7 | 0.3 |
| 32. | Had an Abortion | 6 | 0.3 |
| 33. | Was Raped | 0 | 0.0 |
| 34. | Gave Up a Baby for Adoption | 0 | 0.0 |
| 35. | Lost a Child | 0 | 0.0 |

**CES-D Items, each framed to inquire about past seven days.**

Responses rated as *Never, Rarely, Sometimes*, and *Often*.

1. You were bothered by things that usually don't bother you.
2. You could not shake off the blues, even with help from your family and your friends.
3. You felt you were just as good as other people.
4. You had trouble keeping your mind on what you were doing.
5. You felt depressed.
6. You felt that you were too tired to do things.
7. You felt happy.
8. You enjoyed life
9. You felt sad.
