# Supplementary material for: A Putatively Functional Polymorphism in the HTR2C Gene is Associated with Depressive Symptoms in White Females Reporting Significant Life Stress
Source: PLoS One. 2014 Dec 16;9(12):e114451. doi: 10.1371/journal.pone.0114451 (PMC4267787; doi:10.1371/journal.pone.0114451)
Supplement: S2 Appendix — Spearman correlations among study variables. (DOCX) [file pone.0114451.s002.docx]

**Appendix S2.**

**Spearman Correlations among study variables**

|  | CESD | Stress Index | Antidepressant Medication | Age |
| --- | --- | --- | --- | --- |
| CESD |  | **.280** | **.152** | .030 |
| Stress Index | **.345** |  | **.086** | **-.048** |
| Antidepressant Medication | **.180** | **.086** |  | .012 |
| Age | -.0003 | .011 | .005 |  |

Bolded values indicate p < .05
