# Supplementary material for: A Putatively Functional Polymorphism in the HTR2C Gene is Associated with Depressive Symptoms in White Females Reporting Significant Life Stress
Source: PLoS One. 2014 Dec 16;9(12):e114451. doi: 10.1371/journal.pone.0114451 (PMC4267787; doi:10.1371/journal.pone.0114451)
Supplement: S3 Appendix — Mixed model results for women and men. (DOCX) [file pone.0114451.s003.docx]

**Appendix S3. MIXED MODEL RESULTS**

**WOMEN: PRIMARY MODEL PREDICTING CESD (Square root)**

| **Solution for Fixed Effects** | | | | | | | |
| --- | --- | --- | --- | --- | --- | --- | --- |
| **Effect** | **rs6318** | **Antidepressant Medication** | **Estimate** | **Standard Error** | **DF** | **t value** | **p-value** |
| **Intercept** |  |  | 2.29 | 0.31 | 124 | 7.42 | <.0001 |
| **Antidepressant Medication** |  | 1 | 0.47 | 0.05 | 2580 | 8.49 | <.0001 |
| **Antidepressant Medication** |  | 0 | reference | . | . | . | . |
| **Stress Index** |  |  | 0.16 | 0.01 | 2580 | 14.2 | <.0001 |
| **Age** |  |  | 0.00 | 0.01 | 2580 | -0.1 | 0.922 |
| **rs6318** | C/C |  | -0.11 | 0.13 | 2580 | -0.82 | 0.413 |
| **rs6318** | G/C |  | -0.16 | 0.06 | 2580 | -2.91 | 0.004 |
| **rs6318** | G/G |  | reference | . | . | . | . |
| **Stress Index*rs6318** | C/C |  | 0.14 | 0.07 | 2580 | 2.04 | 0.042 |
| **Stress Index*rs6318** | G/C |  | 0.04 | 0.02 | 2580 | 2.04 | 0.042 |
| **Stress Index*rs6318** | G/G |  | reference | . | . | . | . |

Estimate = mixed model regression coefficient

DF = degrees of freedom

| **Type 3 Tests of Fixed Effects** | | | | |
| --- | --- | --- | --- | --- |
| **Effect** | **Num DF** | **Den DF** | **F value** | **p-value** |
| **Antidepressant Medication** | 1 | 2580 | 72.11 | <.0001 |
| **Stress Index** | 1 | 2580 | 89.26 | <.0001 |
| **Age** | 1 | 2580 | 0.01 | 0.922 |
| **rs6318** | 2 | 2580 | 4.35 | 0.013 |
| **Stress Index*rs6318** | 2 | 2580 | 3.82 | 0.022 |

Num DF = Numerator degrees of freedom

Den DF = Denominator degrees of freedom

| **Least Squares Means (square root CESD)** | | | | | | | | |
| --- | --- | --- | --- | --- | --- | --- | --- | --- |
| **Effect** | **rs6318** | **Stress Index** | **Age** | **Fitted CESD** | **Standard Error** | **DF** | **t value** | **p-value** |
| **rs6318** | C/C | 1 | 28.64 | **2.21** | 0.102 | 2580 | 21.67 | <.0001 |
| **rs6318** | G/C | 1 | 28.64 | **2.06** | 0.045 | 2580 | 46.03 | <.0001 |
| **rs6318** | G/G | 1 | 28.64 | **2.18** | 0.034 | 2580 | 63.98 | <.0001 |
| **rs6318** | C/C | 3 | 28.64 | **2.81** | 0.152 | 2580 | 18.42 | <.0001 |
| **rs6318** | G/C | 3 | 28.64 | **2.47** | 0.045 | 2580 | 54.41 | <.0001 |
| **rs6318** | G/G | 3 | 28.64 | **2.50** | 0.034 | 2580 | 74.66 | <.0001 |

| **Differences of Least Squares Means (square root CESD)** | | | | | | | | |
| --- | --- | --- | --- | --- | --- | --- | --- | --- |
| **Effect** | **rs6318 Genotype Comparison** | **Stress Index** | **Age** | **Difference in CESD** | **Standard Error** | **DF** | **t value** | **p-value** |
| **rs6318** | C/C vs. G/C | 1 | 28.64 | **0.15** | 0.106 | 2580 | 1.43 | 0.154 |
| **rs6318** | C/C vs. G/G | 1 | 28.64 | **0.03** | 0.102 | 2580 | 0.29 | 0.773 |
| **rs6318** | G/C vs. G/G | 1 | 28.64 | **-0.12** | 0.044 | 2580 | -2.77 | 0.006 |
| **rs6318** | C/C vs. G/C | 3 | 28.64 | **0.34** | 0.156 | 2580 | 2.17 | 0.030 |
| **rs6318** | C/C vs. G/G | 3 | 28.64 | **0.30** | 0.153 | 2580 | 1.98 | 0.048 |
| **rs6318** | G/C vs. G/G | 3 | 28.64 | **-0.04** | 0.045 | 2580 | -0.79 | 0.432 |

**MEN: PRIMARY MODEL PREDICTING CESD (Square root)**

| **Solution for Fixed Effects** | | | | | | | |
| --- | --- | --- | --- | --- | --- | --- | --- |
| **Effect** | **rs6318** | **Antidepressant Medication** | **Estimate** | **Standard Error** | **DF** | **t value** | **p-value** |
| **Intercept** |  |  | 1.44 | 0.340 | 122 | 4.23 | <.0001 |
| **Antidepressant Medication** |  | 1 | 0.56 | 0.084 | 2238 | 6.63 | <.0001 |
| **Antidepressant Medication** |  | 0 | reference | . | . | . | . |
| **Stress Index** |  |  | 0.12 | 0.010 | 2238 | 12.54 | <.0001 |
| **Age** |  |  | 0.03 | 0.011 | 2238 | 2.31 | 0.021 |
| **rs6318** | C/- |  | -0.01 | 0.077 | 2238 | -0.09 | 0.931 |
| **rs6318** | G/- |  | reference | . | . | . | . |
| **Stress Index*rs6318** | C/- |  | -0.02 | 0.025 | 2238 | -0.72 | 0.471 |
| **Stress Index*rs6318** | G/- |  | reference | . | . | . | . |

| **Type 3 Tests of Fixed Effects** | | | | |
| --- | --- | --- | --- | --- |
| **Effect** | **Num DF** | **Den DF** | **F value** | **p-value** |
| **Antidepressant Medication** | 1 | 2238 | 43.99 | <.0001 |
| **Stress Index** | 1 | 2238 | 85.62 | <.0001 |
| **Age** | 1 | 2238 | 5.32 | 0.021 |
| **rs6318** | 1 | 2238 | 0.01 | 0.931 |
| **Stress Index*rs6318** | 1 | 2238 | 0.52 | 0.471 |

| **Least Squares Means (square root CESD)** | | | | | | | | |
| --- | --- | --- | --- | --- | --- | --- | --- | --- |
| **Effect** | **rs6318** | **Stress Index** | **Age** | **Fitted CESD** | **Standard Error** | **DF** | **t value** | **p-value** |
| **rs6318** | C/- | 1 | 28.86 | **2.01** | 0.070 | 2238 | 28.58 | <.0001 |
| **rs6318** | G/- | 1 | 28.86 | **2.04** | 0.048 | 2238 | 42.30 | <.0001 |
| **rs6318** | C/- | 3 | 28.86 | **2.22** | 0.065 | 2238 | 34.32 | <.0001 |
| **rs6318** | G/- | 3 | 28.86 | **2.28** | 0.046 | 2238 | 49.72 | <.0001 |

| **Differences of Least Squares Means (square root CESD)** | | | | | | | | |
| --- | --- | --- | --- | --- | --- | --- | --- | --- |
| **Effect** | **rs6318 Genotype Comparison** | **Stress Index** | **Age** | **Difference in CESD** | **Standard Error** | **DF** | **t value** | **p-value** |
| **rs6318** | C/- vs. G/- | 1 | 28.86 | **-0.024** | 0.061 | 2238 | -0.4 | 0.691 |
| **rs6318** | C/- vs. G/- | 3 | 28.86 | **-0.060** | 0.054 | 2238 | -1.1 | 0.271 |
